# Epistasis and Entropy

**DOI:** 10.1371/journal.pgen.1006322

**Published:** 2016-12-22

**Authors:** Kristina Crona

**Affiliations:** American University, Washington, D.C., United States of America; University of Michigan, UNITED STATES

## Abstract

Epistasis is a key concept in the theory of adaptation. Indicators of epistasis are of interest for large systems where systematic fitness measurements may not be possible. Some recent approaches depend on information theory. We show that considering shared entropy for pairs of loci can be misleading. The reason is that shared entropy does not imply epistasis for the pair. This observation holds true also in the absence of higher order epistasis. We discuss a method for reducing the number of false positives. However, our main conclusion is that entropy-based approaches have serious limitations in this context.

## Author Summary

Some recent approaches for identifying epistasis from sequence data depend on information theory. We show that considering shared entropy for pairs of loci can be misleading. The reason is that shared entropy does not imply epistasis for the pair. This observation holds true also in the absence of higher order epistasis. We discuss a method for reducing the number of false positives in the proposed method. However, our main conclusion is that shared entropy for pairs of loci is difficult to interpret. Gene frequencies reflect interactions in the entire system, and there is no natural way to decompose frequency data.

## On Recent Approaches to Entropy and Epistasis

Methods for inferring gene epistasis, or gene interactions, without fitness measurements are valuable for many reasons. It may be difficult or costly, if even possible, to accurately measure fitness for populations in nature.

One approach depends on information theory. Briefly, entropy can be used for finding nonrandom associations for individuals in a population [1]. For instance, if two mutations tend to co-occur, then they will have nonzero shared entropy. Gupta and Adami [2] consider entropy for HIV drug resistance mutations and interpret shared entropy for a pair of mutations as evidence for pairwise epistasis. However, the authors’ conclusion is not valid under realistic assumptions. One cannot deduce pairwise epistasis from shared entropy. Shared entropy may have other causes. For instance, it is well established that a particular mutation (or substitution) may serve as a door opener for new mutations [3, 4]. The new mutations are selected for only if the first mutation has occurred. Such constraints are known from antimicrobial drug resistance, and they may explain many cases of parallel evolution.

In concrete terms, suppose that A and B are drug resistance mutations, but that B is selected for only if A has occurred. This would be the case if the beneficial effect of B depends on the presence of A. In a list of clinically found drug resistance mutants, B would not appear unless A is present (such patterns are far from rare for resistance mutations).

The connection to entropy should be clear. Suppose that another mutation, C, depends on A as well. In that case it is quite plausible that B and C tend to co-occur in the population, and an analysis would reveal nonzero shared entropy for B and C. However, the fitness effects of B and C may be completely independent; i.e., there is no epistasis for B and C.

It is easy to see how misleading the shared entropy condition for pairwise epistasis can be. Suppose that ten mutations are mutually independent (the presence of the others is neither an advantage nor a disadvantage) but that they all depend on A. Then one would identify shared entropy for 55 pairs although there is epistasis for 10 pairs only.

The shared entropy condition is misleading also for the most simple systems. Example 1 in S1 Text concerns a system with no higher order epistasis, i.e, no epistasis beyond pairwise interactions. Two mutations in the system have shared entropy, although their fitness effects are independent. Indeed, 2-way epistasis was excluded both according the geometric classification of gene interactions [5] and according to an approach that depends on Walsh coefficients [6].

Moreover, for a slightly more involved case where the starting point is a heterogeneous population (see Example 3 in S1 Text and the subsequent discussion) the shared entropy is as high as could be for two mutations, although there is no epistasis for the pair.

However, the starting point in Gupta and Adami [2] is sound. If there is no epistasis at all in a system, then one would measure little or no shared entropy for mutations under ideal circumstances. The question is if one can learn anything more specific about epistasis from shared entropy.

Our analysis of the simple rule that shared entropy for two loci implies epistasis for the pair revealed problems. The rule gives false positives. A method that filters out some false positives is discussed in S1 Text. The method depends on considering the entire network of pairs with shared entropy, so as to distinguish between direct and indirect causes for shared entropy. However, the method will not provide a complete solution to the problem of relating epistasis and entropy. Gene interactions are difficult to analyze from frequency data.

Nevertheless, it is possible that some approach of the type proposed in Gupta and Adami [2] could work as a rule of thumb. However, that would require a statistical argument. As it currently stands, there is no foundation for the shared entropy condition for identifying pairwise epistasis.

It should be remarked that the criticism expressed here does not apply to entropy-based methods in human genetics [e.g., 7–9]. Applications of information theory to human genetics depends on the ability to compare genetic information to health conditions. No similar information that directly relates genotype and phenotype is available for the HIV data analyzed in Gupta and Adami [2]. In that sense, the authors considered a more difficult problem.

For a more general perspective, detecting and quantifying epistasis for multilocus systems is a challenging problem, and various new methods have been proposed in recent years. For instance, one line of research provides tools for detecting gene interactions from qualitative data, such as rank orders of genotypes according to fitness [e.g., 10–12].

There is no question that entropy-based approaches, as well as many other recent methods have potential. However, while conducting research in the field we noticed that not all of the methods have been justified by solid theoretical arguments. Moreover, experimentalists have reported seemingly contradicting results from different methods applied to the same protein data (personal communication). Some caution is recommended, and it is probably fair to say that the field is “heroic” rather than “mature” at this point in time.

## Supporting Information

S1 Text (PDF)

## References

[1] Shannon C. E. (1948). A mathematical theory of communication. *Bell System Technical Journal* vol. 27, 379–423 and 623–656, July and October, 1948. 10.1002/j.1538-7305.1948.tb01338.x

[2] Gupta A. and Adami C. (2016). Strong Selection Significantly Increases Epistatic Interactions in the Long-Term Evolution of a Protein. *PLoS Genet* 12(3): e1005960 10.1371/journal.pgen.1005960 27028897 PMC4814079

[3] Beerenwinkel N., Eriksson N. and Sturmfels B. (2007). Conjunctive Bayesian networks. *Bernoulli*; 13:893–909. 10.3150/07-BEJ6133

[4] Desper R., Jiang F., Kallioniemi O.P., Moch H., Papadimitriou C.H. and Schäffer A.A. (1999). Inferring tree models for oncogenesis from comparative genome hybridization data. *Comput. Biol* 6 37–51. 10.1089/cmb.1999.6.37 10223663

[5] Beerenwinkel N., Pachter L. and Sturmfels B. (2007). Epistasis and shapes of fitness landscapes. *Statistica Sinica* 17:1317–1342.

[6] Weinreich D. M., Lan Y., Wily C. S. and Heckendorn R. B. (2013). Should evolutionary geneticists worry about higher-order epistasis? *Curr Opin Gen Dev* 23: 700–7. 10.1016/j.gde.2013.10.007 24290990 PMC4313208

[7] Dong C., Chu X., Wang Y., Wang Y. and Jin L. (2008). Exploration of gene-gene interaction effects using entropy-based methods. *Eur. J. Hum. Genet.* 16: 229–235. 10.1038/sj.ejhg.5201921 17971837

[8] Kang G., Yue W., Zhang J, Cui Y., Zuo Y. and Zhang D. (2008). An entropy-based approach for testing genetic epistasis underlying complex diseases, *J. Theor. Biol.* 250: 362–374. 10.1016/j.jtbi.2007.10.001 17996908

[9] Hu T., Chen Y., Kiralis J.W., Collins R.L., Wejse C., Sirugo G., Williams S.M. and Moore J.H. (2013). An information-gain approach to detecting three-way epistatic interactions in genetic association studies. *J Am Med Inform Assoc.* 10.1136/amiajnl-2012-001525 23396514 PMC3721169

[10] Crona K., Greene D. and Barlow M. (2013). The peaks and geometry of fitness landscapes. *J. Theor. Biol.* 317: 1–13. 10.1016/j.jtbi.2012.09.028 23036916 PMC3529755

[11] Poelwijk FJ, Tanase-Nicola S, Kiviet DJ and Tans SJ. (2011). Reciprocal sign epistasis is a necessary condition for multi-peaked fitness landscapes. *J. Theor Biol.* 272: 141–144. 10.1016/j.jtbi.2010.12.015 21167837

[12] Weinreich D., M, Watson R. A. and Chao L. (2005). Sign epistasis and genetic constraint on evolutionary trajectories. *Evolution.*, 9(6)1165–74. 16050094

