# Supplementary material for: Epistasis and Entropy
Source: PLoS Genet. 2016 Dec 22;12(12):e1006322. doi: 10.1371/journal.pgen.1006322 (PMC5178987; doi:10.1371/journal.pgen.1006322)
Supplement: S1 Text — (PDF) [file pgen.1006322.s001.pdf]

SUPPORTING INFORMATION

BACKGROUND

Epistasis tends to be prevalent for antimicrobial drug resistance mutations. For instance, there are sometimes constraints on the order in which resistance mutations occur. A particular resistance mutation may only be selected for in the presence of another resistance mutation. Epistasis, may be important for treatment strategies, both for antibiotic resistance and HIV drug resistance [3, 4, 13, 16]. Identifying epistasis is therefore a fundamental step in medical applications.

A first question is how one can identify pairwise epistasis in a large system. We will discuss entropy [1] and epistasis. Information theory has been used for HIV drug resistance mutations [2] and more extensively for analyzing human genetic disease [e.g. 7–9]. For recent review articles on epistasis and fitness landscapes see e.g. [14, 15, 18, 19].

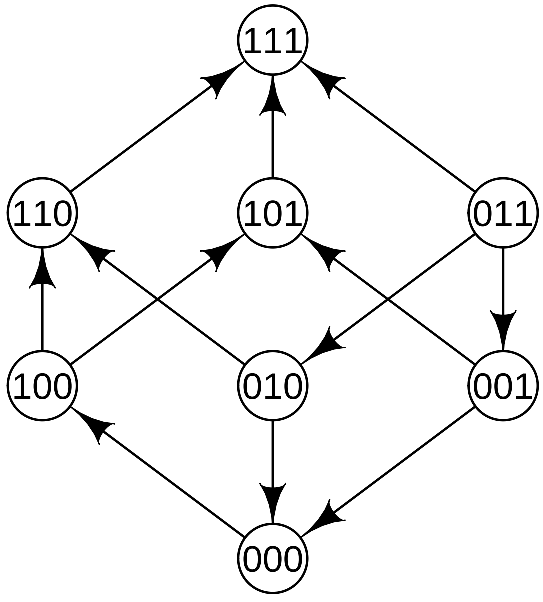

FIGURE 1. The fitness graph for the 3-locus system described in Example 1.

We define fitness as the logarithm of Wrightian fitness and assume that fitness is additive in the absence of epistasis. It is well established that genotypes are expected to be in equilibrium proportions in the absence of epistasis under ideal circumstances. (Physical linkage and other factors can also cause dependence, but ideally the allele frequencies at different loci are independent.) For instance, if two rare mutations have frequencies p and q, then the frequency of the genotype combining the two mutations is expected to be close to pq. This statement holds true regardless if recombination occurs or not [17].

We will explore the relation between entropy and epistasis for a system with constraints as described in the main text. Consider a 3-locus biallelic system where a mutation at the first locus confers resistance, whereas mutations at the second and third loci are only selected for in the presence of the first mutation (otherwise they are deleterious). We represent the case with a fitness graph [10] (Figure 1). As conventional, 000 denotes the wild-type.

For instance, one obtains a system with the fitness graph as in Figure 1 for the following fitness landscape.

Example 1. *Consider the 3-locus system with fitness values as follows.*

$$w_{000} = 0, \quad w_{100} = 0.09531018, \quad w_{010} = -2.302585, \quad w_{001} = -2.302585,$$
$$w_{110} = 0.1906204, \quad w_{101} = 0.1906204, \quad w_{011} = -4.60517, \quad w_{111} = 0.2859305.$$

The gene interactions for a 3-locus system can be described by the sign pattern of 20 circuits, or minimal dependence relations [5]. The relevant two-way interactions in this context be described by the six circuits corresponding to the faces of the 3-cube.

For Example 1 the circuit sign pattern is:

$$\begin{aligned}
w_{000} - w_{010} - w_{100} + w_{110} &> 0 \\
w_{001} - w_{011} - w_{101} + w_{111} &> 0 \\
w_{000} - w_{001} - w_{100} + w_{101} &> 0 \\
w_{010} - w_{011} - w_{110} + w_{111} &> 0 \\
w_{000} - w_{001} - w_{010} + w_{011} &= 0 \\
w_{100} - w_{101} - w_{110} + w_{111} &= 0.
\end{aligned}$$

The four inequalities express that there is positive epistasis for the first and second loci, as well as for the first and third loci. The two equalities show that there is no epistasis for the second and third loci, regardless of background. The total 3-way epistasis is zero as well,

$$w_{111} - w_{110} - w_{101} - w_{011} + w_{100} + w_{010} + w_{001} - w_{000} = 0.$$

Higher order gene interactions have also been described using Walsh coefficients [6]. For this landscape the Walsh coefficient $E_{011} = 0$, which indicates an absence of background averaged epistasis for the second and third loci.

We will consider entropy during the process of adaptation for this landscape. The starting point for adaptation is the wild-type 000. We use a standard Wright-Fisher model for an infinite population with mutation rate $\mu = 10^{-7}$. The gene frequencies and shared entropy after the given number of generations are listed in the table.

TABLE 1. A simulation of the 3-locus system in Example 1. The table shows the gene frequencies and shared entropy $I(2,3)$ for an infinite population with mutation rate 10^{-7}.

generations	000	100	010	001	110	101	011	111	I(2,3)
130	0.7692	0.1850	0	0	0.0214	0.0214	0	0.0031	0.003206041
140	0.4834	0.3015	0	0	0.0904	0.0904	0	0.0343	0.01736237
146	0.2723	0.3008,	0	0	0.1597	0.1597	0	0.1075	0.02335234
150	0.1569	0.2539	0	0	0.1974	0.1974	0	0.1944	0.0211462
160	0.0229	0.0959	0	0	0.1934	0.1934	0	0.4943	0.006950302
170	0.0020	0.0216	0	0	0.1132	0.1132	0	0.7501	0.001270666

The shared entropy for the second and third loci differs from zero. However, there is no 2-way epistasis for the pair of loci. It is intuitively clear why the shared entropy differs from zero. Indeed, the epistasis expressed by the four inequalities implies that a mutation at the second locus (from 0 to 1) benefits from the presence of a mutation at the first locus (the first coordinate being 1), and the same is true for a mutation at the third locus.

Example 2. *By extrapolation, consider an analogous system analogous to Example 1 for L loci. Specifically, $L-1$ mutations are selected for only if the first mutation has occurred, but there are no other interactions.*

In Example 2 there is non-zero shared entropy for $\binom{L}{2}$ pairs of loci, although there is 2-way epistasis for $L-1$ pairs of loci only. For instance, for 20 loci one finds 190 pairs of loci with non-zero shared entropy, although there is epistasis for 19 pairs only.

A pair with no epistasis and maximal shared entropy. The following fitness landscapes is closely related to Example 1.

Example 3. *Consider the 3-locus system with fitness values as follows.*

$$w_{000} = 0, \quad w_{100} = 0, \quad w_{010} = -2.302585, \quad w_{001} = -2.302585,$$
$$w_{110} = 0.09531018, \quad w_{101} = 0.09531018, \quad w_{011} = -4.60517, \quad w_{111} = 0.1906204.$$

This example is closely related to the Example 1. Indeed, the two-way interactions can be described by the sign pattern

$$\begin{aligned}
w_{000} - w_{010} - w_{100} + w_{110} &> 0 \\
w_{001} - w_{011} - w_{101} + w_{111} &> 0 \\
w_{000} - w_{001} - w_{100} + w_{101} &> 0 \\
w_{010} - w_{011} - w_{110} + w_{111} &> 0 \\
w_{000} - w_{001} - w_{010} + w_{011} &= 0 \\
w_{100} - w_{101} - w_{110} + w_{111} &= 0,
\end{aligned}$$

and the total 3-way epistasis is zero:

$$w_{111} - w_{110} - w_{101} - w_{011} + w_{100} + w_{010} + w_{001} - w_{000} = 0.$$

Also in this case, there is no epistasis for the second and third loci. Mutations at the second and third loci are selected for only in the presence of a mutation at the first locus. However, the critical difference is that a mutation at the first locus is neutral for the wild-type in Example 3.

Suppose that 50 percent of hosts start a new treatment with 000 viruses, and 50 percent start with the 100 genotype. That could be realistic, for instance if the 100 genotype had some resistance to a previously used drug. By assumption, eventually one would have about 50 percent 000 genotypes and 50 percent 111 genotype in the total population. Then $I(2,3) = 1$ although there is no epistasis for the second and third loci. This example also points at a fundamental problem relating pairwise epistasis and entropy. At the time when we have 50 percent 000 genotypes and 50 percent 111 genotypes, obviously no method can reveal pairwise epistasis.

A method for reducing the number of false positives. If one interprets shared entropy for pairs of loci as an indication of epistasis [2] one gets false positives, as we have seen. We will discuss an approach for reducing the number of false positives. Suppose that we have identified shared entropy for a particular pair of loci $\{k, l\}$.

Let $S_1^{k,l}$ denote the set of loci such that the shared entropy

$$I(k : i) \neq 0 \text{ or } I(l : i) \neq 0.$$

Let $S_2^{k,l}$ denote the set of loci with non-zero shared entropy for some locus in $S_1^{k,l}$, and so forth. Let $S^{k,l} = \bigcup S_i \setminus \{k, l\}$.

Let v denote one of the $2^{|S|}$ possible states for S, and consider the subsystem of genotypes determined by v. If the shared entropy $I^v(k : l) = 0$ for all v, then there is no indication of of epistasis for $\{l, k\}$.

We can apply the method on Example 3, specifically the case discussed where the end result was 50 percent 000 and 50 percent 111 genotypes, and $I(2,3) = 1$. Then

$$S = \{1\}, \quad I^{(0)}(2:3) = I^{(1)}(2:3) = 0.$$

Consequently, there is no indication of epistasis for the second and third loci.

The described method could be useful for identifying some cases with shared entropy and no epistasis. It remains to explore to what extent the method is useful in other settings.

DISCUSSION

We have demonstrated that shared entropy for two loci does not imply epistasis for the pair. This observation holds true also in the absence of 3-way epistasis in a single environment. We discussed a method which filters out some cases where shared entropy depends on states at other loci. However, our main conclusion is that shared entropy for pairs of loci is difficult to interpret. Gene frequencies reflect interactions in the entire system, and there is no natural way to decompose frequency data.

There are obviously other reasons for caution in interpretations of entropy for drug resistance mutations. Different drugs constitute different environments. Some resistance mutations at different loci may be correlated if they are beneficial in the presence of a particular drug, but not for other drugs. In such cases entropy would not not imply epistasis.

Our results show that observations on entropy and epistasis based on 2-locus systems can be misleading for general systems. From a theoretical point of view, a better understanding of large systems would be useful for handling drug resistance data.

METHODS

Let x and y be discrete random variables with states x_1, x_2 and y_1, y_2. Let p_i denote the frequency of x_i, and p_{ij} the frequency for the combination of x_i and y_j. The entropy [1] $H(x)$ and the joint entropy $H(x,y)$ are defined as

$$\begin{aligned} H(x) &= -\,p_1 \log(p_1) - (1-p_1)\log(1-p_1) \\ H(x,y) &= -\,p_{11}\log p_{11} - p_{12}\log(p_{12}) \\ &\quad - p_{21}\log p_{21} - p_{22}\log(p_{22}). \end{aligned}$$

The shared entropy is the quantity $I(x:y) = H(x) + H(y) - H(x,y)$.

In general $I(x:y) \geq 0$, and the shared entropy is a measure of dependence.

REFERENCES

1. Shannon, C. E. (1948). A mathematical theory of communication. *Bell System Technical Journal* vol. 27, 379–423 and 623-656, July and October, 1948.
2. Gupta, A. and Adami, C. (2016). Strong Selection Significantly Increases Epistatic Interactions in the Long-Term Evolution of a Protein. *PLoS Genet* 12(3): e1005960. doi:10.1371/journal.pgen.1005960.
3. Beerenwinkel, N., Eriksson, N. and Sturmfels, B. (2007). Conjunctive Bayesian networks. *Bernoulli*; 13:893–909.

4. Desper, R., Jiang, F., Kallioniemi, O.P., Moch, H., Papadimitriou, C.H. and Schäffer, A.A. (1999). Inferring tree models for oncogenesis from comparative genome hybridization data. *Comput. Biol* 6 37–51.
5. Beerenwinkel, N., Pachter, L. and Sturmfels, B. (2007). Epistasis and shapes of fitness landscapes. *Statistica Sinica* 17:1317–1342.
6. Weinreich D. M., Lan, Y., Wily, C. S. and Heckendorn, R. B. (2013) . Should evolutionary geneticists worry about higher-order epistasis? *Curr Opin Gen Dev* 23: 700-7.
7. Dong, C., Chu, X., Wang, Y., Wang, Y. and Jin, L. (2008). Exploration of gene-gene interaction effects using entropy-based methods. *Eur. J. Hum. Genet.* 16: 229-235.
8. Kang, G., Yue, W., Zhang, J, Cui,Y., Zuo, Y. and Zhang, D. (2008). An entropy-based approach for testing genetic epistasis underlying complex diseases, *J. Theor. Biol.* 250: 362–374.
9. Hu, T., Chen, Y., Kiralis, J.W., Collins, R.L., Wejse, C., Sirugo, G., Williams, S.M. and Moore, J.H. (2013). An information-gain approach to detecting three-way epistatic interactions in genetic association studies. *J Am Med Inform Assoc.*
10. Crona, K., Greene, D. and Barlow, M. (2013). The peaks and geometry of fitness landscapes. *J. Theor. Biol.* 317: 1–13.
11. Poelwijk FJ, Tanase-Nicola S, Kiviet DJ and Tans SJ. (2011). Reciprocal sign epistasis is a necessary condition for multi-peaked fitness landscapes. *J. Theor Biol.* 272: 141–144.
12. Weinreich, D. M, Watson, R. A. and Chao, L. (2005). Sign epistasis and genetic constraint on evolutionary trajectories. *Evolution.*, 9(6)1165-74.
13. Goulart, C. P., Mentar, M., Crona, K., Jacobs, S. J., Kallmann, M., Hall, B. G., Greene D. and Barlow M. (2013). Designing antibiotic cycling strategies by determining and understanding local adaptive landscapes. *PLoS ONE* 8(2): e56040. doi:10.1371/journal.pone.0056040.
14. Hartl, D. (2014) What can we learn from fitness landscapes? *Current Opinion in Microbiology* 21 (2014): 51-57.
15. Kondrashov, D. A. and Kondrashov , F. A. (2014). Topological features of rugged fitness landscapes in sequence space. *Trends in Genetics*.
16. Mira, P. M., Crona, K., Greene, D., Meza. J.C., Sturmfels, B. and Barlow, M. (2015). Rational Design of Antibiotic Treatment Plans: A Treatment Strategy for Managing Evolution and Reversing Resistance. *PLoS ONE* 10(5): e0122283. doi:10.1371/journal.pone.0122283.
17. Otto, S. P. and Lenormand, T. (2002). Resolving the paradox of sex and recombination. *Nature Reviews Genetics*; 3:252-261.
18. Szendro, I. G., Schenk, M. F., Franke, J. Krug, J. and de Visser J. A. G. M. (2013). Quantitative analyses of empirical fitness landscapes *J. Stat. Mech. P01005.*
19. De Visser, J. A. G. M, and Krug, J. (2014) Empirical fitness landscapes and the predictability of evolution." *Nature Reviews Genetics* 15.7 (2014): 480-490.
